# Structural and mechanistic basis of the high catalytic activity of monooxygenase Tet(X4) on tigecycline

**DOI:** 10.1186/s12915-021-01199-7

**Published:** 2021-12-11

**Authors:** Qipeng Cheng, Yanchu Cheung, Chenyu Liu, Qingjie Xiao, Bo Sun, Jiahai Zhou, Edward Wai Chi Chan, Rong Zhang, Sheng Chen

**Affiliations:** 1 State Key Lab of Chemical Biology and Drug Discovery, Department of Applied Biology and Chemical Technology, The Hong Kong Polytechnic University, Hung Hom, Kowloon, Hong Kong; 2 Department of Infectious Diseases and Public Health, Jockey Club College of Veterinary Medicine and Life Sciences, City University of Hong Kong, Kowloon, Hong Kong; 3 Shanghai Advanced Research Institute, Chinese Academy of Sciences, No.239 Zhangheng Road, Shanghai, 201204 China; 4 CAS Key Laboratory of Quantitative Engineering Biology, Shenzhen Institute of Synthetic Biology, Shenzhen Institute of Advanced Technology, Chinese Academy of Sciences, Shenzhen, 518055 China; 5 Department of Clinical Laboratory, Second Affiliated Hospital of Zhejiang University, School of Medicine, Hangzhou, 310009 People’s Republic of China

**Keywords:** Tet(X4), Variant, Tigecycline, FAD binding, Secondary structure

## Abstract

**Background:**

Tigecycline is a tetracycline derivative that constitutes one of the last-resort antibiotics used clinically to treat infections caused by both multiple drug-resistant (MDR) Gram-negative and Gram-positive bacteria. Resistance to this drug is often caused by chromosome-encoding mechanisms including over-expression of efflux pumps and ribosome protection. However, a number of variants of the flavin adenine dinucleotide (FAD)-dependent monooxygenase TetX, such as Tet(X4), emerged in recent years as conferring resistance to tigecycline in strains of Enterobacteriaceae, *Acinetobacter* sp., *Pseudomonas* sp., and *Empedobacter* sp. To date, mechanistic details underlying the improvement of catalytic activities of new TetX enzymes are not available.

**Results:**

In this study, we found that Tet(X4) exhibited higher affinity and catalytic efficiency toward tigecycline when compared to Tet(X2), resulting in the expression of phenotypic tigecycline resistance in *E. coli* strains bearing the *tet*(X4) gene. Comparison between the structures of Tet(X4) and Tet(X4)-tigecycline complex and those of Tet(X2) showed that they shared an identical FAD-binding site and that the FAD and tigecycline adopted similar conformation in the catalytic pocket. Although the amino acid changes in Tet(X4) are not pivotal residues for FAD binding and substrate recognition, such substitutions caused the refolding of several alpha helixes and beta sheets in the secondary structure of the substrate-binding domain of Tet(X4), resulting in the formation of a larger number of loops in the structure. These changes in turn render the substrate-binding domain of Tet(X4) more flexible and efficient in capturing substrate molecules, thereby improving catalytic efficiency.

**Conclusions:**

Our works provide a better understanding of the molecular recognition of tigecycline by the TetX enzymes; these findings can help guide the rational design of the next-generation tetracycline antibiotics that can resist inactivation of the TetX variants.

**Supplementary Information:**

The online version contains supplementary material available at 10.1186/s12915-021-01199-7.

## Background

The abusive usage of antibiotics in the past few decades resulted in widespread drug resistance, a clinical and public health problem that poses a significant threat to human health. As a last-resort antibiotic, tigecycline was approved for clinical use by FDA in 2005 and is still effective against multiple-resistant (MDR) pathogens [1, 2]. However, following increased usage of this antibiotic, resistant strains have emerged [3–5]. Previously, tigecycline resistance mechanisms mainly involve activities of non-specific efflux pumps and ribosomal protection [6]. Recently, several plasmid-encoded variants of the tetracycline-degrading enzyme Tet(X), such as Tet(X4) which confers high-level tigecycline resistance, were found to be produced by MDR bacteria isolated from animals and humans [7–12]. These novel enzymes degrade tetracyclines more effectively than Tet(X). Organisms carrying genetic elements that encode these enzymes have been disseminated in clinical and veterinary practices, prompting a public health concern. Despite intensive research to investigate why new TetX variants exhibited higher catalytic activity towards tigecycline, the detailed mechanism involved remains poorly understood.

In this study, we characterized the mechanism underlying the degradation of tigecycline by Tet(X4), resolved the crystal structure of the Tet(X4) and tigecycline-Tet(X4) complex, and revealed the substrate basis of Tet(X4)-mediated catalysis. Compared to the structure of Tet(X2), the secondary structure of the substrate-binding domain of Tet(X4) deconstructed a large number of α-helix and β-sheet which resulted in the loss of various internal contact points in the structure, rendering the substrate-binding domain more flexible in allowing access of the tigecycline molecule to flavin adenine dinucleotide (FAD) for oxidation.

## Results

### Tet(X4) against tetracycline antibiotics

Consistent with previous studies [8, 10, 13], *E. coli* strain BW25113 carrying the plasmid pBAD-18-*tet*(X4) was found to exhibit an 8–64-fold increase in MIC of various tetracycline antibiotics when compared with the host strain (Table 1). We observed the in vitro degradation of tigecycline by purified recombinant Tet(X4) (Fig. S1), which was characterized by a time-dependent decrease in the 350~420-nm absorbance in the UV absorbance spectrum due to breakage of the conserved β-diketone chromophore in the tigecycline (Fig. S2) [7].

**Table 1:** Susceptibility of tetracyclines in *E. coli* BW25113 harboring a pBAD18 vector which contains the *tet*(X2), *tet*(X4), or mutated *tet*(X2) gene

*E. coli* BW25113 strains	MIC (mg/L)		
	Tetracycline	Minocycline	Tigecycline
Vector control	2	2	0.25
29522	1	0.25	0.125
Tet(X2)	32	4	1
Tet(X4)	64	16	16
L^282^S	32	8	4
V^329^M	32	8	2

To explore the substrate binding and catalytic efficiency of Tet(X4), an enzyme kinetic assay was performed on Tet(X4) by continuously monitoring the decrease in UV absorbance at 400 nm under steady-state conditions. We also purified Tet(X2) and used it as a control by determining the kinetic parameters of Tet(X2) toward tigecycline. The tigecycline-degrading efficiency of Tet(X4) was about 4.8 folds higher than that of Tet(X2), with the *k*_cat_*/K*_*M*_ values being 1.13×10^6^ M^−1^ s^−1^ and 2.33×10^5^ M^−1^ s^−1^, respectively (Table 2). This difference in catalytic efficiency is due to both increase in substrate turnover (*k*_*cat*_) and higher substrate-binding affinity (*K*_*M*_) by Tet(X4) when compared to Tet(X2). The increased activity of Tet(X4) toward tigecycline is similar to that of the previously reported tigecycline resistance-conferring enzyme Tet(X7) [7]. The inactivation of tigecycline by Tet(X4) was also analyzed by ESI-mass spectrometry; the primary product of tigecycline was observed at peak *m*/*z* 586.4 in all reactions (Fig. S3). A new product peak at *m*/*z* 602.5 was detected upon incubation of Tet(X4) with tigecycline for 30 min, which is corresponding to the addition of one oxygen atom to tigecycline (*m*/*z* 586.5), suggesting that Tet(X4) is likely a monooxygenase.

**Table 2:** Kinetic parameters of Tet(X2), Tet(X4), and enzymes carrying the L^282^S or V^329^M substitutions on tigecycline

Protein	*k*_cat_ (S^−1^)	*K*_*M*_ (μM)	*k*_cat_/*K*_*M*_ (M^−1^ S^−1^)
Tet(X2)	1.04±0.01	4.45±0.13	2.33×10^5^
Tet(X4)	2.03±0.03	1.80±0.09	1.13×10^6^
L^282^S	1.36±0.05	3.96±0.38	3.43×10^5^
V^329^M	3.65±0.10	4.81±0.32	7.59×10^5^

### Structure of Tet(X4)

**Fig. 1.**
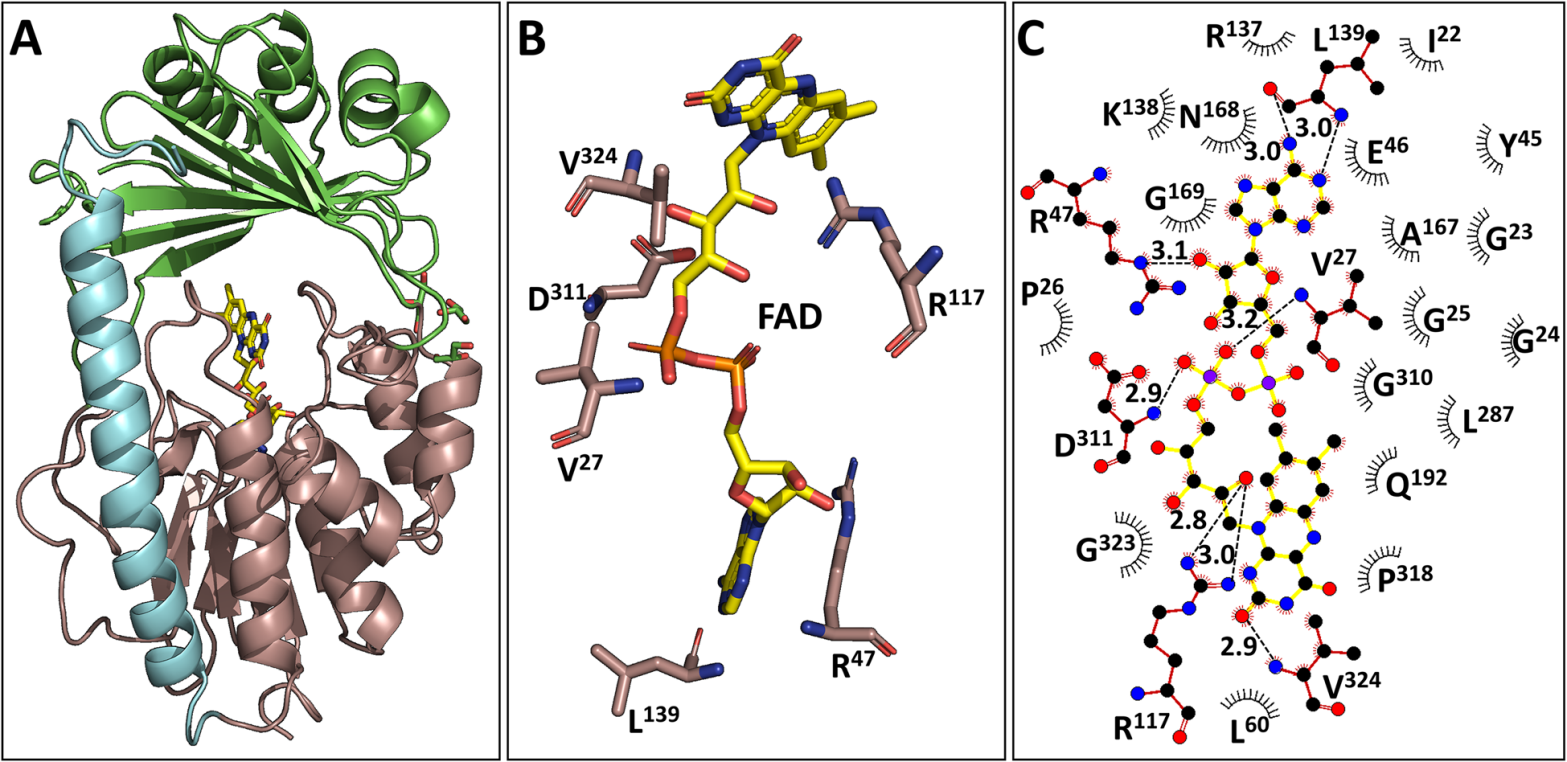
Crystal structure of Tet(X4). **A** Overall structure of Tet(X4) has a conserved FAD-binding motif (dark salmon), a substrate-binding domain (green), and a C-terminal bridge helix (cyan). **B** The FAD-binding site of Tet(X4), FAD is shown in yellow, and protein carbon atoms are depicted in dark salmon. **C** Interaction between FAD and Tet(X4). FAD is shown in yellow. Relevant hydrogen bonds are shown as dashed lines and their distances are expressed in Å. The figure was generated by LigPlot+ [14]

We solved the X-ray crystal structure of Tet(X4) at a resolution of 1.78 Å, with the key dataset and refinement statistics being shown in Table S1. It was found to exhibit a typical folding pattern similar to that of the previously reported tetracycline destructase, with a FAD-binding domain, a substrate-binding domain, and a C-terminal α-helix bridging the two domains (Fig. 1). Its structure was shown to be almost identical (root-mean-square deviation [RMSD] of 0.35 Å and 0.38 Å, respectively) to that of other members of tetracycline destructase, such as Tet(X2) (PDB: 2XDO, with 96.04% sequence identity) from *Bacteroides thetaiotaomicron* and Tet(X7) (PDB: 6WG9, with 88.89% sequence identity) from *Pseudomonas aeruginosa*. Only one monomer was observed in the crystallographic asymmetric unit in all structures. The monomer includes 369 amino acids of Tet(X4) (Asn^247^, Gln^248^, and Thr^249^ were not modeled), namely, Asn^12^ to Gln^383^. The final R factor and R free values of the refined structures varied from 16.63 to 18.53% and 19.97 to 23.88%, respectively.

**Fig. 2.**
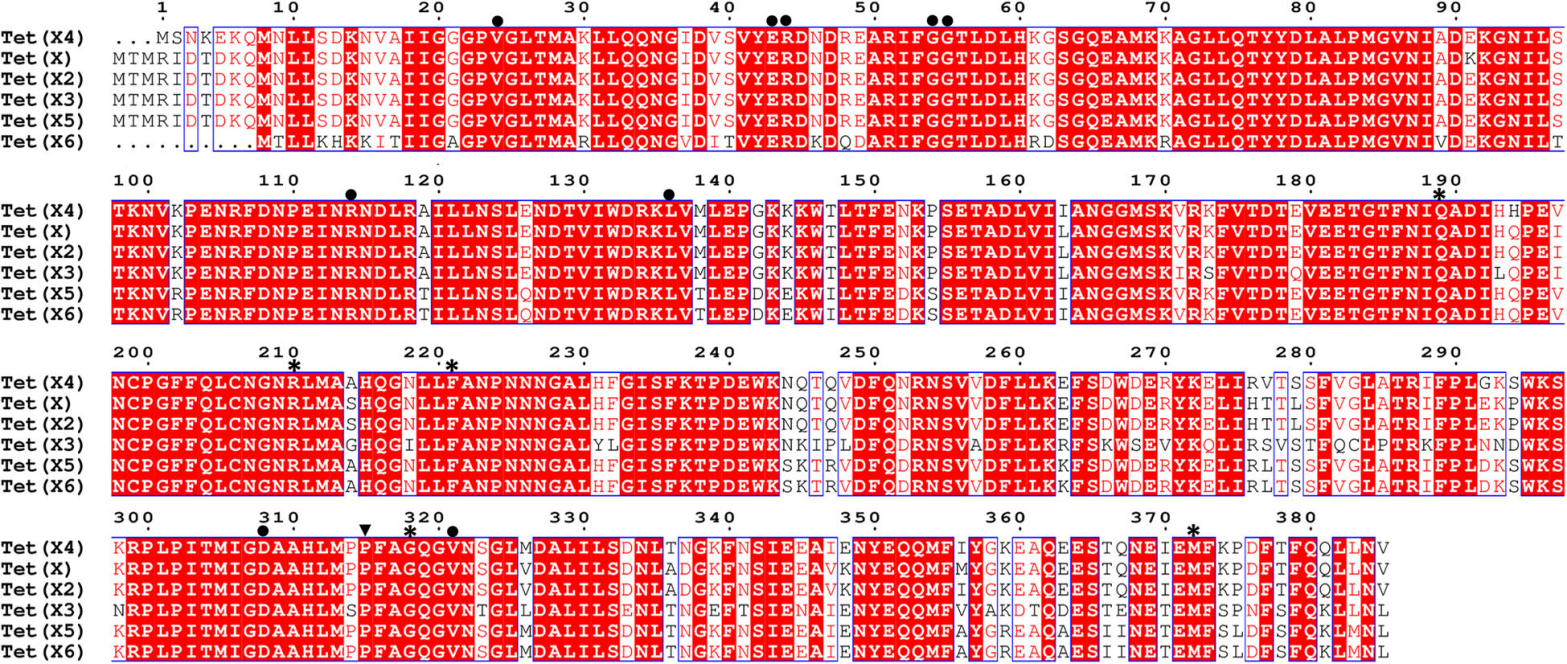
Alignment of the protein sequence of Tet(X4) with other TetX variants. The strictly conserved amino acid residues are boxed in red. Physicochemically similar amino acids are shown in red. FAD-binding sites are indicated by a solid circle (•); substrate recognition sites are indicated by black stars (*); residue P^318^ is the key site for FAD and substrate binding and is depicted as a solid inverted triangle (▼). The figure was prepared using CLUSTAL Omega [16] and ESPript [17]

**Fig. 3.**
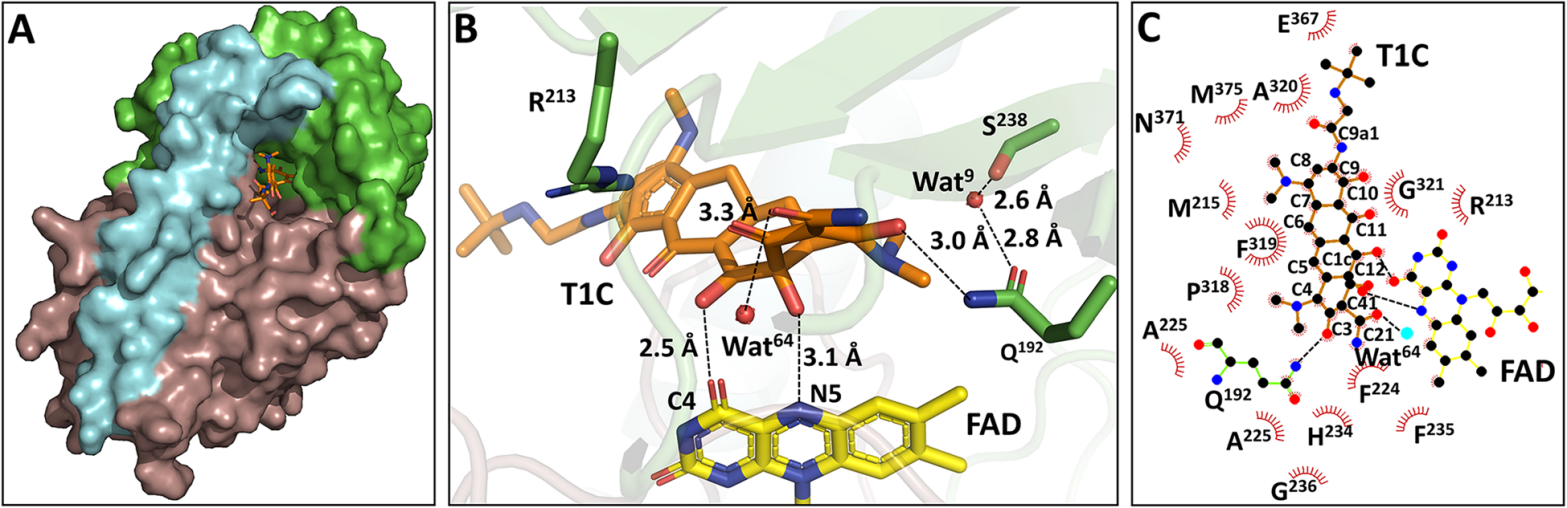
Overview of the process by which tigecycline binds to Tet(X4). **A** Surface representation of the tigecycline (T1C) binding cavity. **B** Active site recognition of T1C by hydrogen-bonding of Tet(X4) (protein residues in green, T1C in orange, FAD in yellow, hydrogen bonds as black dashed lines). **C** Interaction between T1C and Tet(X4). Residues around the binding pocket are depicted as green sticks, T1C is depicted in orange, and FAD is depicted in yellow

In the Tet(X4) and Tet(X4)-tigecycline structures, FAD is bound non-covalently to an IN-conformation such as those of Tet(X) and Tet(X7) (Figs. 1 and 3) [7, 15]. The FAD-binding residues (Val^27^, Glu^46^, Arg^47^, Gly^57^, Gly^58^, Arg^117^, Leu^139^, Asp^311^, Pro^318^, and Val^324^) are conserved in Tet(X2) and Tet(X4). Moreover, multiple sequence alignment (MSA) revealed that these residues are also conserved among all Tet(X) variants (Fig. 2).

### Recognition of tigecycline by Tet(X4)

Accommodation of tigecycline in the binding pocket of Tet(X4) was investigated by X-ray diffraction studies of tigecycline-soaked Tet(X4) crystals. Analysis of the Tet(X4)-tigecycline complex structure revealed specific interactions between the tigecycline hydroxylation product (T1C) and the isoalloxazine of FAD in the large active site cavity (Fig. 3A). Electron density maps allow for unambiguous identification and placement of a T1C molecule in the crystal. In the Tet(X4)-tigecycline complex structure, the FAD cofactor also adopts the IN-conformation analogous to the Tet(X4) structure (Fig. 3A).

**Fig. 4.**
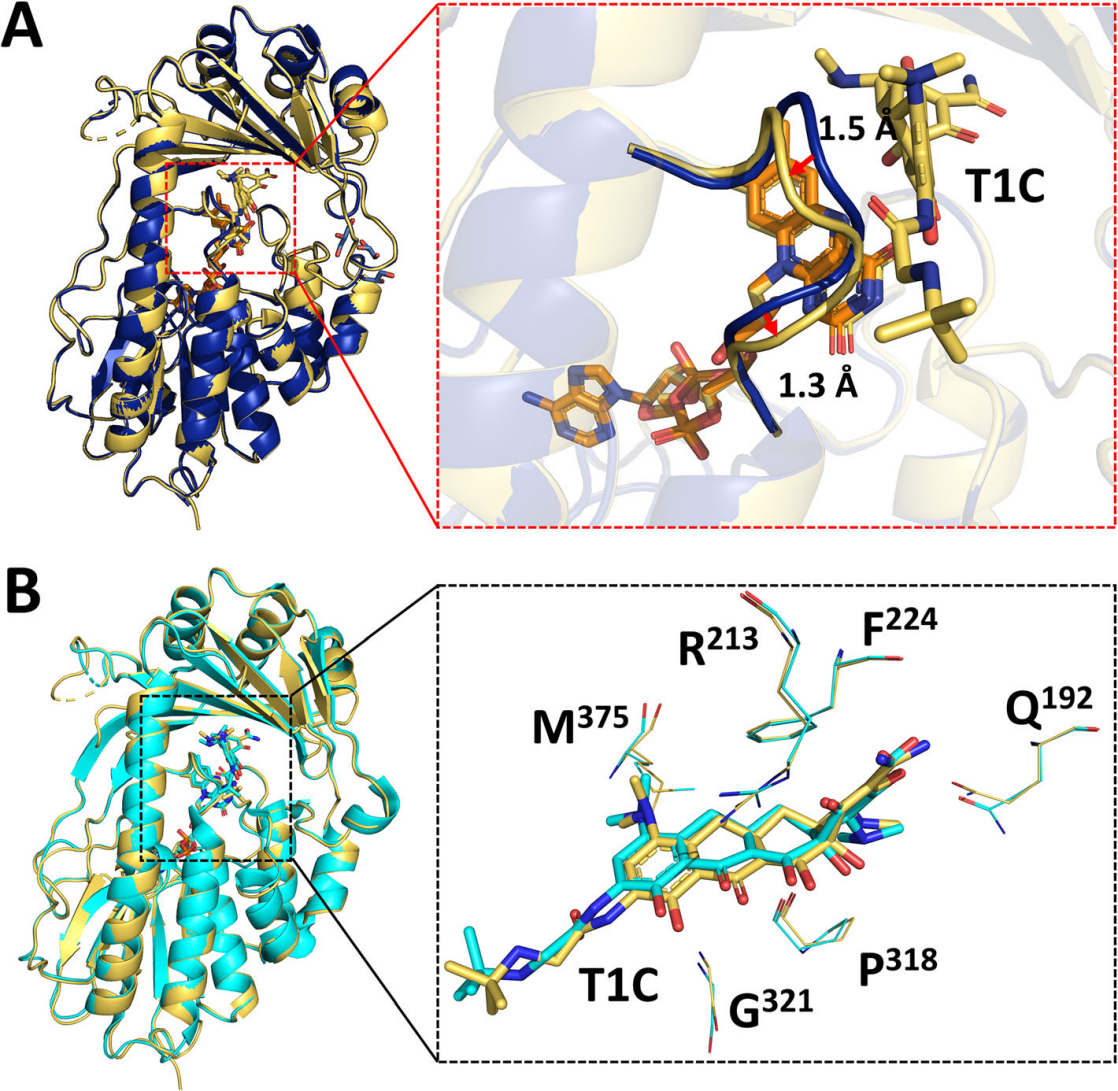
Structural comparison of Tet(X4)-tigecycline complex with Tet(X4) and Tet(X2)-tigecycline complex. **A** Cartoon and superimposition of Tet(X4)-tigecycline structure (the color of the cartoon is shown in yellow) and Tet(X4) structure (the color of the cartoon is shown in blue). The red dashed box indicates the dynamic changes in the loop between α10 and α11, with details shown in the right enlarged dashed box. FAD and T1C are shown as sticks. **B** Superposition of the structure of Tet(X4)-tigecycline complex (the color of the cartoon is shown in yellow) with the Tet(X2)-tigecycline complex (4A6N, the color of the cartoon is shown in cyan). The black dashed box indicates the interactions of TIC with Tet(X4) and Tet(X2), with details shown in the right enlarged dashed box. T1C are shown as sticks; conserved interacting residues are depicted as lines

The A ring of T1C forms hydrogen bonds with the side chain of Gln^192^ (Fig. 3B, C). A water molecule (Wat^9^) establishes a hydrogen-bonding bridge connecting the hydroxyl group of Ser^238^ to the carbonyl O atom of Gln^192^; this type of interaction was also observed in the TetX-minocycline complex structure [18]. Like the previously reported Tet(X)-tetracycline complex structures [15, 18, 19], the cofactor FAD in the deep cavity of Tet(X4) was also found to form hydrogen bonds with the hydroxyl groups at C1c and C12 of T1C via the N^5^ and O^4^ atoms (Fig. 3B, C), which plays a key role in stabilizing substrate binding. Another water molecule (Wat^64^) also forms a hydrogen bond (3.2 Å) with the hydrophilic region (C21) of T1C (Fig. 3B, C). The hydrophilic sites Arg^213^, Ala^225^, H^234^, Ala^320^, G^321^, Glu^367^, and Asn^371^ were found to be able to interact (3.0–4.0 Å) with the hydroxyl sites at C11, C21, C10, and C91 of T1C. Since T1C does not have any specific group at C5, C6, C8, and C9, only the functional group of C7 makes van der Waals contacts with the side chains of Met^215^, Asn^371^, and Met^375^ (within 4.0 Å). Furthermore, the hydrophobic segment of T1C (C41–C7) forms several interactions with the side chains of hydrophobic residues Phe^224^, Pro^318^, and Phe^319^. These observations indicate that recognition of tigecycline by the active site of Tet(X4) mainly involves targeting of the conserved hydrophilic substituents in the A ring and C10, C11, and C12 of T1C. The Tet(X4)-tigecycline complex structure is highly identical to the Tet(X2)-tigecycline complex structure, with RMSD at 0.4 Å. T1C adopts similar conformations that interact with Gln^192^, Arg^213^, Phe^224^, Pro^318^, Gly^321^, and Met^375^, which is observable in both the Tet(X4)-tigecycline and Tet(X2)-tigecycline complex structures (Fig. 4). Such features are also detectable in other tetracycline-antibiotic complex structures [15, 18, 19].

**Fig. 5.**
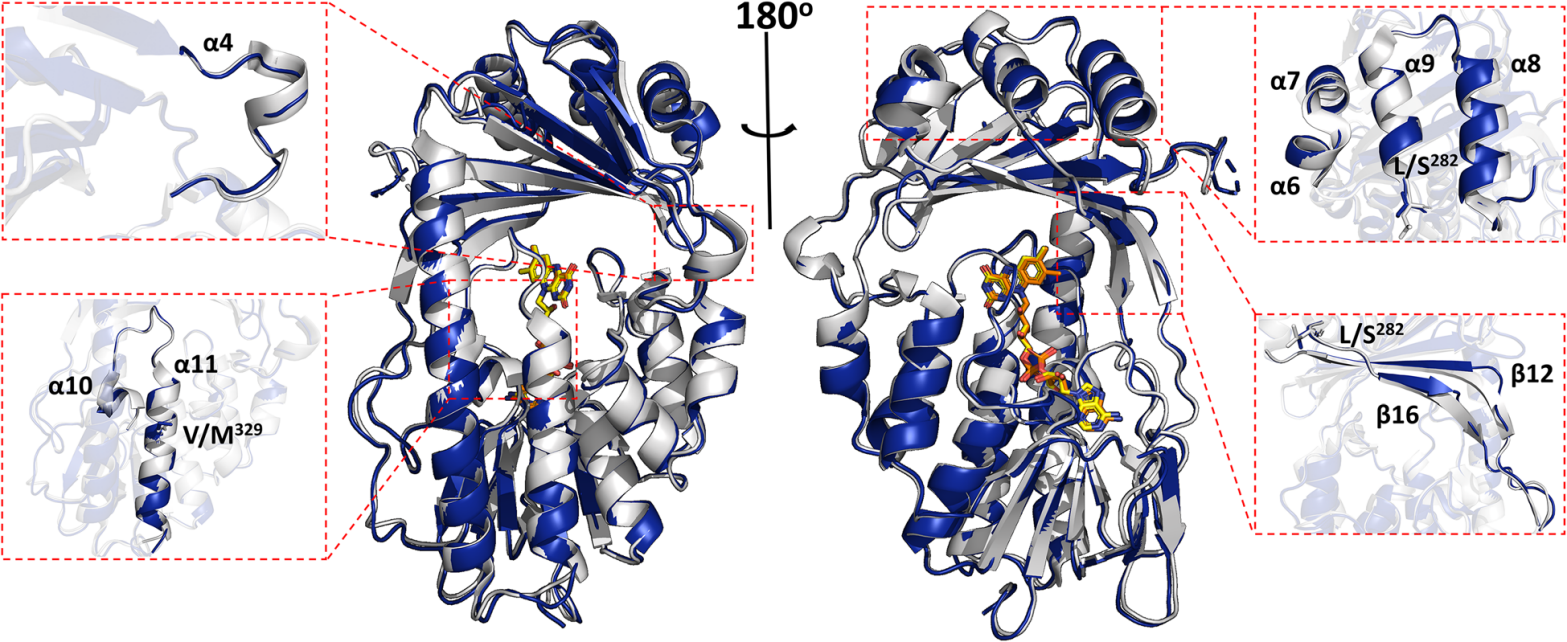
Superimposition of the Tet(X4) structure (cartoon of Tet(X4) shown in blue) and Tet(X2) structure (cartoon of Tet(X2) shown in gray). FAD in Tet(X4) is shown in orange, FAD in Tet(X2) is shown in yellow; the red dashed box indicates the structural difference between the substrate-binding domain of Tet(X4) and Tet(X2), with details shown in the enlarged dashed box; key residues of Tet(X4) that are different from Tet(X2) are depicted as sticks

The RMSD of the Cβ positions between the tigecycline complex structure and Tet(X4) is 0.1 Å, suggesting that insertion of the tigecycline molecule did not cause dramatic conformational changes in the Tet(X4) monooxygenase structure (Fig. 4A). Upon capturing of tigecycline by Tet(X4), the loop between α10 and α11 exhibited a dynamic shift in position when compared to the structure of Tet(X4), with Cα of Ala^320^ and Gln^322^ being shifted 1.3 Å and 1.5 Å, respectively (Figs. 4A and 5). These changes might expand the cavity and facilitate contact with FAD. Furthermore, when compared to Tet(X4) structure, the value of the B-factor of the loop (H^314^-G^323^) was found to have increased from 22.6 to 52.9 Å^2^ in the Tet(X4)-tigecycline complex structure, indicating that the loop exhibits higher mobility for ligand binding and release. This observation also shows that the loop (H^314^-G^323^) plays an important role in substrate recognition by Tet(X4).

### Secondary structure changes in the substrate-binding domain of Tet(X4)

Superimposition of the structure of Tet(X4) and structure of Tet(X2) revealed the difference between the substrate-binding domain of the two enzymes (Fig. 5). First, α10 and α11 in the Tet(X4) structure were shorter when compared to Tet(X2), indicating that the loop between α10 and α11 was extended to allow accommodation of a more flexible conformation. The loop has been considered the substrate-binding site; hence, an extended loop might enhance the chance by which tigecycline attaches to FAD for oxidization. In addition, a number of alpha helixes in Tet(X4), namely α6, α7, α8, and α9, have collapsed to generate more loops for substrate binding (Fig. 5). α4 formed an alpha helix structure in the Tet(X2) structure, but it was deconstructed as a loop in Tet(X4) structure (Fig. 5). As the main components of the substrate-binding domain, β12 and β16 in Tet(X4) have also become smaller in size, forming a larger number of loops when compared to Tet(X2) (Fig. 5). These structural alterations allowed the secondary structure of Tet(X4) to adopt more loops and turns, rendering the structure of the substrate-binding domain more flexible and more readily to accommodate the tetracycline molecule, resulting in increased catalytic efficiency of enzyme on tigecycline. These structural features therefore explain the phenotype resistance of Tet(X4)-producing organisms.

The changes in the secondary structure of Tet(X4) have been supported by CD scan and FTIR analysis (Table 3, Figs. S4 and S5). CD spectroscopic studies revealed that the composition of α-helix (29.3%) and β-sheet (24.7%) of Tet(X4) have slightly decreased by 2–3% when compared to Tet(X2). FTIR analysis of Tet(X4) protein solution, which indicated that the proportion of helices (α-helix and 3_10_-helix) and β-sheet declined to 30.8% and 40.4%, respectively (Table 3), is consistent with the results of CD experiments. All in all, it was evident that the percentage of α-helix and β-sheet has decreased significantly in Tet(X4), rendering the secondary structure of Tet(X4) more flexible.

**Table 3:** Comparison of the relative content (%) of different types of secondary structure in Tet(X4), Tet(X2), and enzymes harboring the L^282^S and V^329^M substitution

	CD analysis				FTIR analysis			
	Tet(X2)	Tet(X4)	L^282^S	V^329^M	Tet(X2)	Tet(X4)	L^282^S	V^329^M
α-helix (%)	32.4	29.3	31.6	26.9	17.1	16.4	15.5	14.9
3_10_-helix (%)	–	–	–	–	26.7	14.4	11.8	11.4
β-sheet (%)	26.1	24.7	23.9	23.9	50	40.4	43.8	50.7
Others (%)	41.5	46	44.5	49.2	6.8	28.8	28.9	23
Spectral deviation (RMSD)	0.182	0.132	0.123	0.109	–	–	–	–

### Amino acid substitutions cause changes in the secondary structure of Tet(X4)

Compared to Tet(X2), the variant residues in Tet(X4) are not in vital sites for FAD binding and substrate recognition, but such amino acid changes might drastically affect the secondary structure of Tet(X4). To confirm our hypothesis, we introduced L^282^S or V^329^M into Tet(X2) by site-directed mutagenesis. Residues 282 connected α9 and β16 (Fig. 5); when the amino acid leucine was substituted by serine at position 282, analyses by CD and FTIR both showed that the percentage of α-helix and β-sheet in the L^282^S-bearing protein decreased significantly, indicating that the L^282^S substitution caused a drastic change in the secondary structure of the protein (Figs. S4 and S5, Table 3). In contrast, FTIR analysis showed that the proportion of β-sheet remained steady at 50.7% in the V^329^M-bearing variant enzyme, but the percentage of α helices decreased to 26.3% (Fig. S4, S5, Table 3). As residue 329 is located at α11 (Fig. 5), the amino acid substitution V^329^M might extend the loop between α10 and α11 but has less effect on β-sheet. Furthermore, *E. coli* BW25113 carrying the L^282^S or V^329^M changes exhibited a 2–4-fold increase in MIC when compared to the Tet(X2)-producing strain. Consistently, the tigecycline-catalyzing efficiency of L^282^S and V^329^M also increased 1.5–3.5 folds when compared to Tet(X2). These findings indicate that amino acid substitutions could cause changes in the secondary structures of Tet(X4) and improve the catalytic activity of the enzyme.

## Discussion

Since their discovery in the 1940s, tetracyclines have become the key antimicrobial agents in agricultural, veterinary, and clinical applications [20, 21]. As a result, resistance to tetracycline antibiotics became increasingly observed in both Gram-negative and Gram-positive bacteria. Such resistance phenotypes were found to be caused by efflux activities and ribosome protection [21, 22]. Subsequent advancements in tetracycline modification techniques resulted in the third- and fourth-generation tetracyclines such as tigecycline, eravacycline, and omadacycline, which could effectively combat tetracycline resistance [23–25]. However, the newly discovered plasmid-borne *tet*(X) genes, which encode tetracycline inactivation enzymes such as TetX4, were found to be responsible for conferring resistance to the latest-generation tetracycline antibiotics among MDR Gram-negative pathogens [8, 10]. Furthermore, with increasing selective pressure imposed by the last generation tetracyclines, organisms that carry novel *tet*(X) variants, such as *tet*(X5-14), have emerged and disseminated extensively [7, 9, 11, 26]. However, the structural basis of the increased catalytic activity of Tet(X4) remains unknown.

In this work, we showed that Tet(X4) exhibited a high affinity toward tigecycline and catalyzed tigecycline more efficiently than Tet(X2). These functional properties of Tet(X4) are responsible for causing the phenotype of tigecycline resistance in *E. coli* strains bearing the *tet*(X4) gene. Interestingly, Tet(X4) shared a highly identical FAD-binding site with that of Tet(X2), with tigecycline being accommodated in a similar conformation in the catalytic cavity. Compared to Tet(X2), the amino acid changes in Tet(X4) are not the pivotal residues for FAD-binding and substrate recognition. However, these amino acid substitutions, such as L^282^S and V^329^M, might cause the refolding of several alpha helixes and beta sheets in the secondary structure of the substrate-binding domain of Tet(X4), allowing a larger number of loops to form in the structure. These changes render the substrate-binding domain of Tet(X4) more flexible and efficient in capturing substrates and thereby improving catalysis. Loop regions as the most flexible parts of protein structures often play an important role in protein functions by interacting with the solvent and substrates [27]. The finding that the dynamic change of structure of TetX4 resulted in an enhanced catalytic activity is consistent with that of a previous report on the effect of directed evolution of Tet(X) toward tigecycline [28].

Structure-guided approaches have been proven to be effective in assisting the discovery of antibiotic analogs and inhibitors [29, 30]. The high-resolution structures of Tet(X4) and Tet(X4)-tigecycline complex provided a deep understanding of the molecular recognition mechanism of tigecycline by Tet(X)s enzymes; such knowledge facilitates rational design of novel tetracycline antibiotics that can escape enzymatic inactivation and remain active against a wide range of MDR bacterial pathogens.

## Conclusions

In conclusion, we investigated the structural basis of the high catalytic activity of Tet(X4) on tigecycline, which is one of the last line antibiotics used to treat multidrug-resistant bacterial infections. We found that Tet(X4) exhibited a high affinity toward tigecycline and catalyzed tigecycline more efficiently than Tet(X2). These functional properties were due to carriage of specific amino acid substitutions, such as L^282^S and V^329^M, in Tet(X4) which might cause the refolding of several alpha helixes and beta sheets in the secondary structure of the substrate-binding domain, allowing a larger number of loops to form in the structure. These changes render the substrate-binding domain of Tet(X4) more flexible and efficient in capturing substrates and thereby improving catalysis. These findings facilitate the design of next-generation tetracycline which can resist degradation by Tet(X4).

## Methods

### Bacterial strains and functional cloning of *tet(X)s*

The full-length *tet*(X4) gene was amplified from genomic DNA of ST767 *E. coli* strain [13] by two pairs of primers (Table S2). The PCR product which contained the *EcoR* I /*Sal* I restriction sites was sub-cloned into the plasmid vector pBAD18-kan (kanamycin resistance), which contained the arabinose pBAD promoter. Another *tet*(X4) gene PCR product with the *BamH* I/*Xho* I restriction sites was ligated to a modified pET-M vector which contained 3C protease cleave site beyond the His6 tag. Subsequently, the recombinant plasmid pBAD-18-*tet*(X4) was transformed into competent cells of *E. coli* BW25113, followed by antibiotic susceptibility tests. The recombinant plasmid pET-M-*tet*(X4) was transformed into competent *E. coli* BL21 (DE3) cells for protein expression and purification. We also constructed two *tet*(X2)-bearing vectors as control. Mutations were introduced into the *tet*(X2) gene using the QuickChange Site-Directed Mutagenesis Kit (Stratagene).

### Antimicrobial susceptibility test

Antibiotic susceptibility test of *E. coli* strain BW25113 harboring the *tet*(X4)-bearing pBAD18-kan vector was performed. Minimum inhibitory concentrations (MICs) were determined according to Clinical and Laboratory Standards Institute (CLSI) procedures using Mueller–Hinton broth microdilution method [31]. *E. coli* strain ATCC 25922 was used as quality control.

### Protein expression and purification

0.5 liter of Luria Broth (LB) containing 100 μg/mL ampicillin was inoculated into a 5-mL overnight culture, followed by incubation with shaking at 37 °C until an optical density of 0.6 at 600 nm (OD600) was reached. The expression of enzymes was induced by 0.5 mM isopropyl-β-D-thiogalactopyranoside (IPTG) at 16°C for 16 h. The cells were harvested by centrifugation at 11,300×*g* for 5 min and resuspended in lysis buffer (25 mM Tris-HCl, pH8.0, 300 mM NaCl, 30 mM imidazole, and 1mM PMSF) and then broken by high-pressure homogenization. The soluble fractions were passed through a Ni-nitrilotriacetic acid (NTA) column, rinsed with 25 mM Tris-HCl, pH 8.0, 300 mM NaCl, and 30 mM imidazole, and finally eluted with 25 mM Tris-HCl, pH 8.0, 300 mM NaCl, and 300 mM imidazole. The eluted proteins were concentrated using the Amicon Ultra-15 (nominal molecular weight limit [NMWL] = 30 000) centrifugal filter device. The purified enzyme was incubated with 3C protease at 4 °C overnight to remove the His6 tag. The target proteins were further purified by gel filtration chromatography (Superdex 75; GE Healthcare) in a buffer of 20 mM Tris (pH 7.5), 150 mM NaCl, and 2 mM DTT (Dithiothreitol). The desired fractions were collected and concentrated. The purity of the protein was determined by sodium dodecyl sulfate-polyacrylamide gel electrophoresis (SDS-PAGE) (Fig. S1).

### Steady-state kinetics of Tet(X4)

Each 500-μL reaction was prepared with 100 mM TAPS buffer at pH 8.5 with 0–50 μM substrate, 5 mM MgCl_2_, and 0.5 mM nicotinamide adenine dinucleotide phosphate (NADPH). UV-visible spectroscopy measurements were performed in triplicate at 400-nm wavelength light, using a UV-1900 UV-Vis spectrophotometer (Shimadzu) for measurement for 3 min at room temperature. Initial reaction velocities were determined by linear regression using the UVProbe 2.70 Software and fitted to the Michaelis–Menten equation by GraphPad Prism 8.

### Crystallization, data collection, and structure refinement

Tet(X4) protein was concentrated to 22 mg/mL and crystallized by sitting drop vapor diffusion at 16 °C in 0.2 M ammonium acetate, 0.1 M sodium citrate tribasic dihydrate pH 5.6, and 30% (w/v) polyethylene glycol 4000. Tigecycline was soaked into the crystals by incubating the Tet(X4) crystals in a reservoir buffer for 30 min. The crystals were cryoprotected by 25% glycerol in reservoir buffer for 10 s and flash-cooled in liquid nitrogen. Diffraction data were collected at 100 K on beamline BL17U1 at the Shanghai Synchrotron Radiation Facility [32]. The diffraction data were processed by xds [33], xia2 [34], and aimless [35]. The Tet(X4) structure was solved by molecular replacement using Phaser, with free Tet(X) structure (PDB: 2XYO) as the search model. Structure refinement was performed by using Phenix [36], REFMAC [37], and Coot [38]. The structures have been deposited to PDB as 7EPV and 7EPW (validated by PDB, Additional file 8). The structure figures were prepared by PyMOL [39].

### Fourier transform infrared (FTIR) spectroscopy

Infrared spectra were recorded on a PerkinElmer Spectrum 100 instrument using an attenuated total reflection (ATR) sampling accessory as described in previous studies [40, 41]. Briefly, protein solutions were loaded in the well, and data was acquired at 25 °C in the range of 1700−1600 cm^−1^ (wavenumber). Typically, eight scans were collected and averaged for a single spectrum with a resolution of 4 cm^1^. The background was corrected before scanning the samples. The FITR spectra of Tet(X2), Tet(X4), L^282^S, and V^329^M were collected in a buffer of 20 mM Tris (pH 7.5) and 150 mM NaCl; the absorbance of the buffer was subtracted from the spectra of Tet(X2), Tet(X4), L^282^S, and V^329^M. The concentrations of Tet(X2), Tet(X4), L^282^S, and V^329^M in the assay were 20–30 mg/mL.

### Circular dichroism (CD)

CD spectra were measured by a Jasco J-1500 spectropolarimeter at 25 °C. CD measurements in a spectral range of 260 to 200 nm were performed, with an interval of 1 nm and scanning speed of 50 nm min^−1^ corrected with baseline. The CD spectra of Tet(X2), Tet(X4), L^282^S, and V^329^M protein solutions were acquired, and changes in CD spectral results were analyzed. The secondary structures were determined by BeStSel [42, 43].

## Supplementary Information

**Additional file 1: Figure S1.** Size-exclusion chromatogram of Tet(X4), peak 2 is corresponded to Tet(X4); the inset shows reduced SDS–PAGE analysis of the purified proteins.

**Additional file 2: Figure S2.** (A) Chemical structure of tigecycline. Rings B-D is responsible for β-diketone chromophore that was circled by the orange rectangle. (B) *In vitro* absorbance scan at wavelength between 300nm and 500 nm taken at 60 seconds intervals, covering the Tet(X4) protein, NADPH, MgCl_2_, and tigecycline. The rainbow shape illustrates the spectral change over time. Time-dependent decrease in absorbance from 370 nm to 420 nm indicates enzymatic disruption of the characteristic tigecycline β-diketone chromophore and consumption of NADPH.

**Additional file 3: Figure S3.** Mass spectrometry analysis of enzymatic reactions with tigecycline as substrate. (A), Reaction without enzyme at 0 minutes; (B), Reaction without enzyme at 30 minutes; (C), Reaction of Tet(X4) with tigecycline as substrate at 0 minutes; (D), Reaction of Tet(X4) with tigecycline as substrate at 30 min.

**Additional file 4: Table S1.** Crystallographic data and refinement statistics.

**Additional file 5: Figure S4**. (A) Representative mean spectra of Tet(X2) protein solution shown in the range 1600–1700 cm^-1^ after baseline correction and vectorial normalization; (B) Curve-fitting analysis of amide I band of Tet(X2) protein solution; (C) Representative mean spectra of Tet(X4) protein solution shown in the range 1600–1700 cm^-1^ after baseline correction and vectorial normalization; (D) Curve-fitting analysis of amide I band of Tet(X4) ; (E) Representative mean spectra of the L^282^S mutant protein solution shown in the range 1600–1700 cm^-1^ after baseline correction and vectorial normalization; (F) Curve-fitting analysis of amide I band of the L^282^S mutant protein; (G) Representative mean spectra of the V^329^M mutant protein solution shown in the range 1600–1700 cm^-1^ after baseline correction and vectorial normalization; (H) Curve-fitting analysis of amide I band of the V^329^M mutant protein.

**Additional file 6: Figure S5**. (A) Circular dichroic spectral profiles of Tet(X2) protein solution measured at wavelength between 200 nm and 260 nm; the raw CD spectrum is shown; (B) Circular dichroic spectral profiles of Tet(X4) protein solution measured at wavelength between 200 nm and 260 nm; (C) Circular dichroic spectral profiles of the L^282^S mutant protein solution measured at wavelength between 200 nm and 260 nm; (D) Circular dichroic spectral profiles of the V^329^M mutant protein solution measured at wavelength between 200 nm and 260 nm.

**Additional file 7: Table S2**. Primers were used in this study.

**Additional file 8.** Validation reports of the structure of Tet(X4) and Tet(X4)-tigecycline complex.

## Data Availability

All data generated or analyzed during this study are included in this published article and its supplementary information files. The structures in this study have been deposited to PDB as 7EPV and 7EPW.

## Abbreviations

ATR: Attenuated total reflection
CD: Circular dichroism
CLSI: Clinical and Laboratory Standards Institute
DTT: Dithiothreitol
FAD: Flavin adenine dinucleotide
FTIR: Fourier transform infrared
IPTG: Isopropyl-β-D-thiogalactopyranoside
LB: Luria Broth
MDR: Multiple drug resistant
MICs: Minimum inhibitory concentrations
NADPH: Nicotinamide adenine dinucleotide phosphate
NTA: Ni-nitrilotriacetic acid
RMSD: Root-mean-square deviation
SDS-PAGE: Sodium dodecyl sulfate-polyacrylamide gel electrophoresis
