# Supplementary figures and images for: Structural and mechanistic basis of the high catalytic activity of monooxygenase Tet(X4) on tigecycline
Source: BMC Biol. 2021 Dec 11;19:262. doi: 10.1186/s12915-021-01199-7 (PMC8666040; doi:10.1186/s12915-021-01199-7)

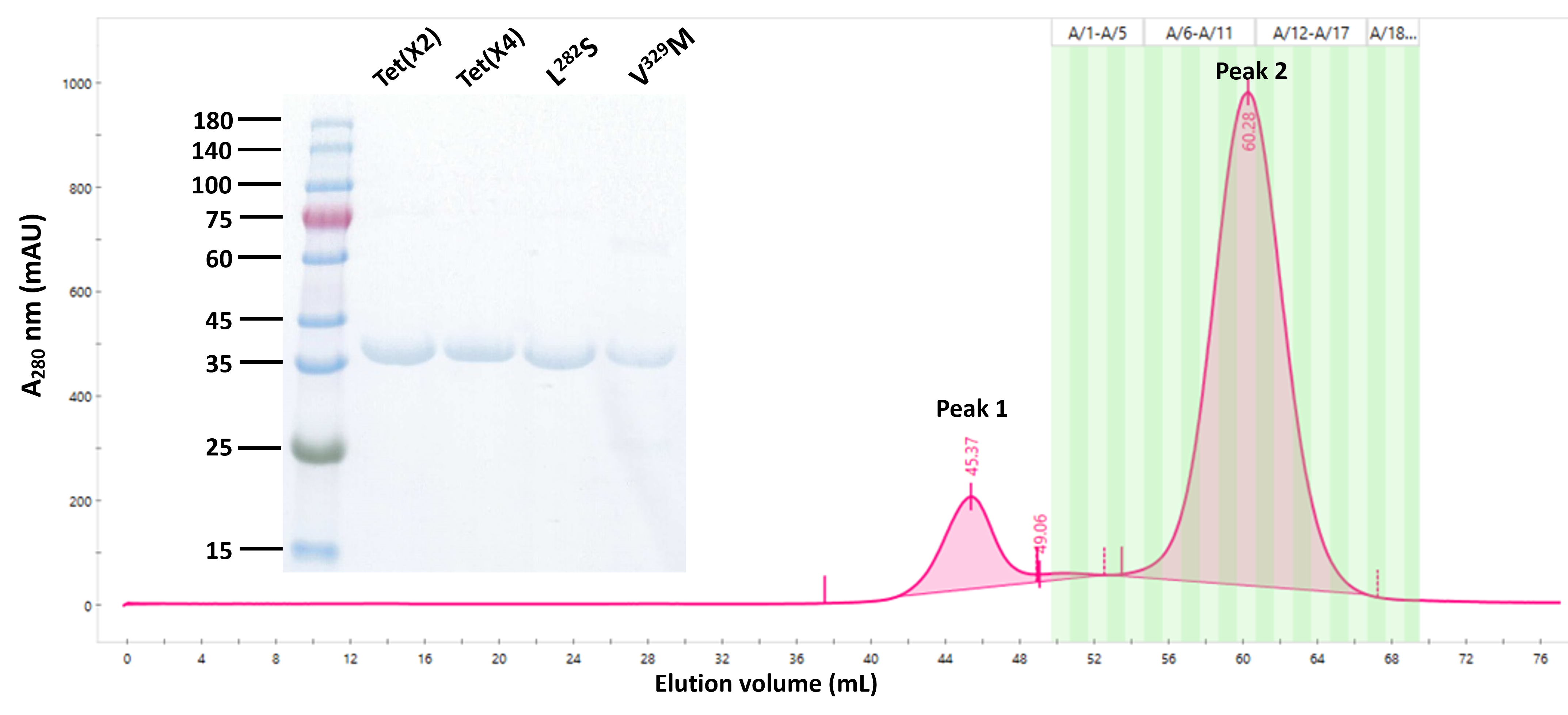

Supplement: Supplementary file 1 — Additional file 1: Figure S1. Size-exclusion chromatogram of Tet(X4), peak 2 is corresponded to Tet(X4); the inset shows reduced SDS–PAGE analysis of the purified proteins. [file 12915_2021_1199_MOESM1_ESM.tif]

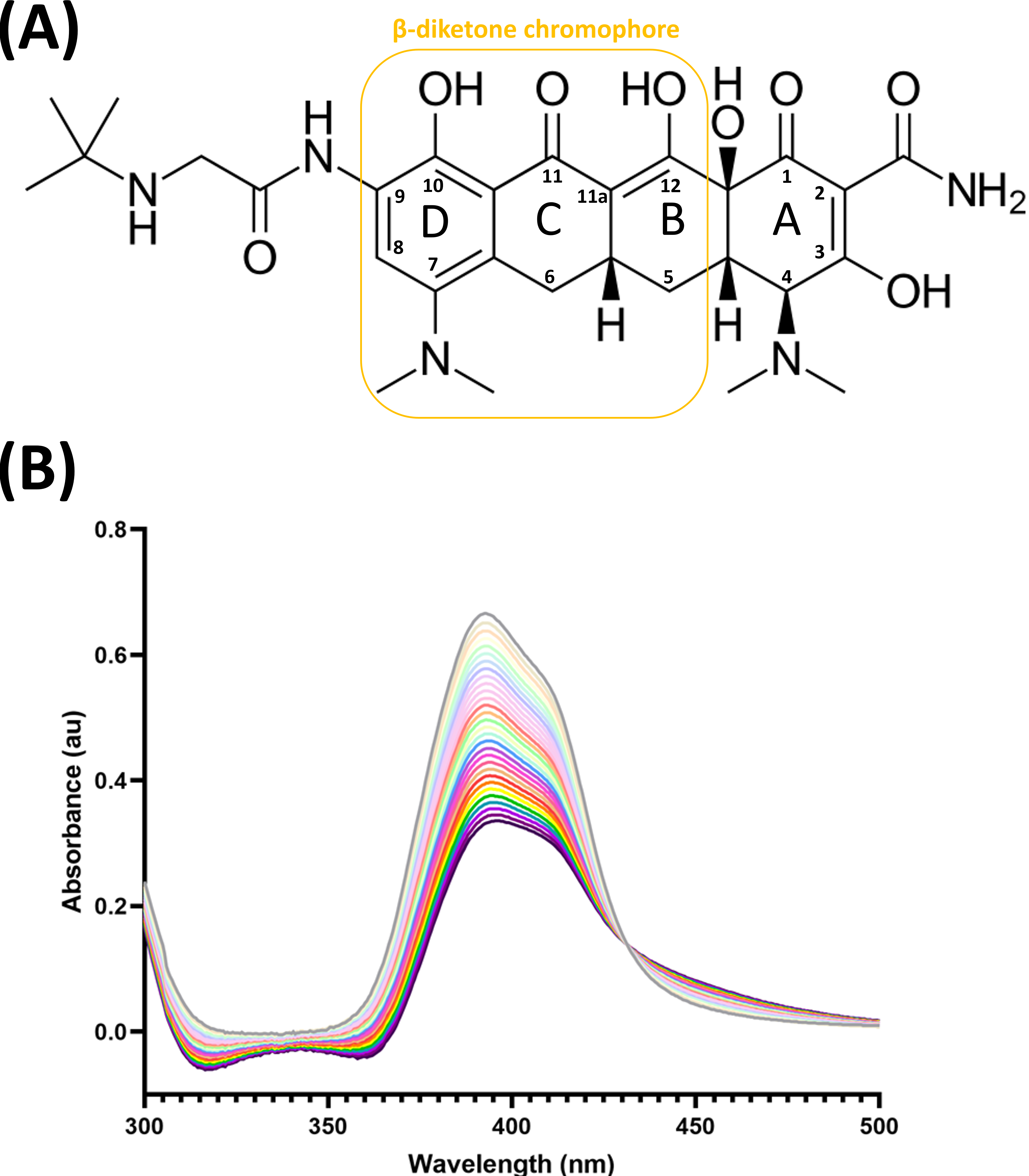

Supplement: Supplementary file 2 — Additional file 2: Figure S2. (A) Chemical structure of tigecycline. Rings B-D is responsible for β-diketone chromophore that was circled by the orange rectangle. (B) In vitro absorbance scan at wavelength between 300nm and 500 nm taken at 60 seconds intervals, covering the Tet(X4) protein, NADPH, MgCl2, and tigecycline. The rainbow shape illustrates the spectral change over time. Time-dependent decrease in absorbance from 370 nm to 420 nm indicates enzymatic disruption of the characteristic tigecycline β-diketone chromophore and consumption of NADPH. [file 12915_2021_1199_MOESM2_ESM.tif]

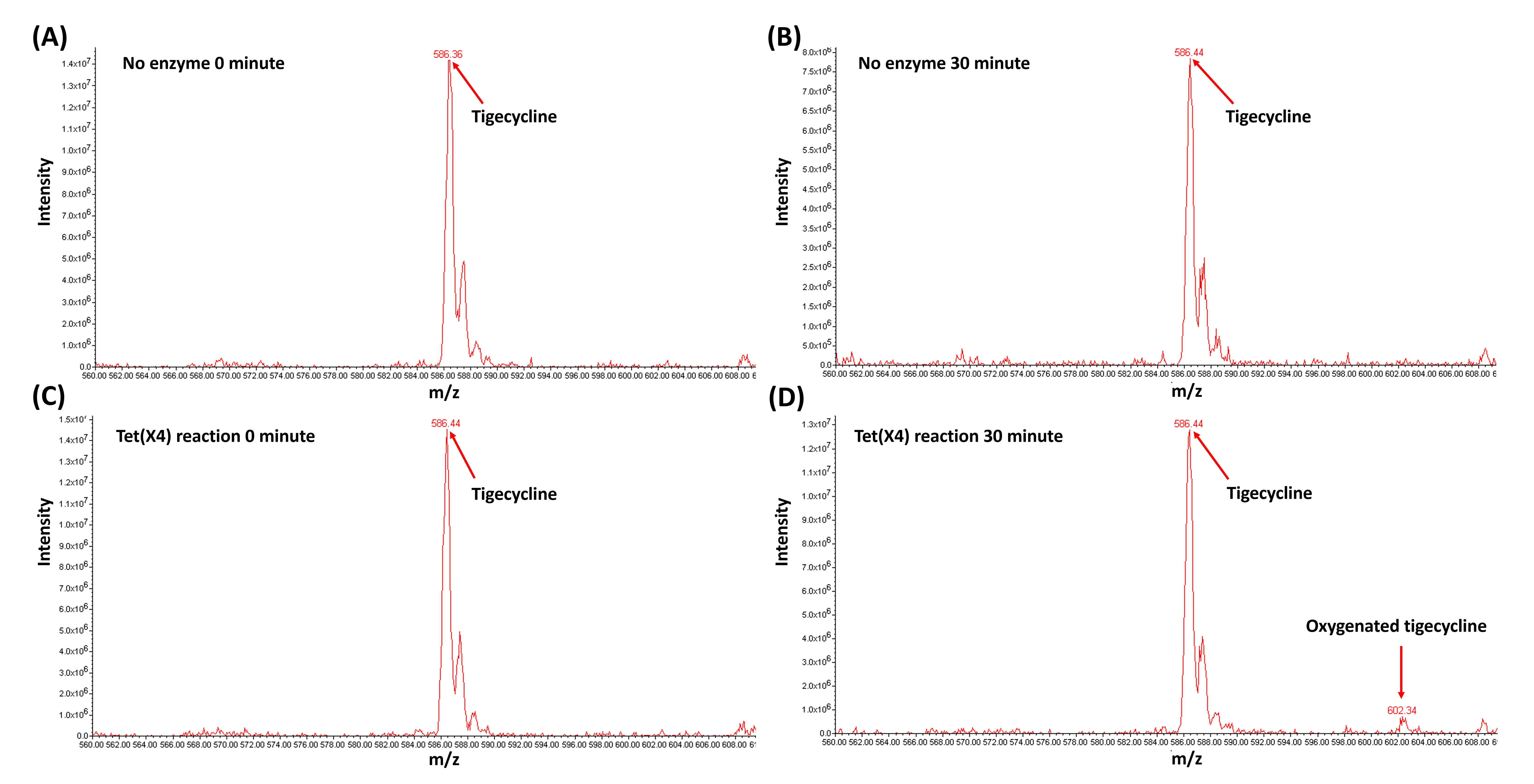

Supplement: Supplementary file 3 — Additional file 3: Figure S3. Mass spectrometry analysis of enzymatic reactions with tigecycline as substrate. (A), Reaction without enzyme at 0 minutes; (B), Reaction without enzyme at 30 minutes; (C), Reaction of Tet(X4) with tigecycline as substrate at 0 minutes; (D), Reaction of Tet(X4) with tigecycline as substrate at 30 min. [file 12915_2021_1199_MOESM3_ESM.tif]

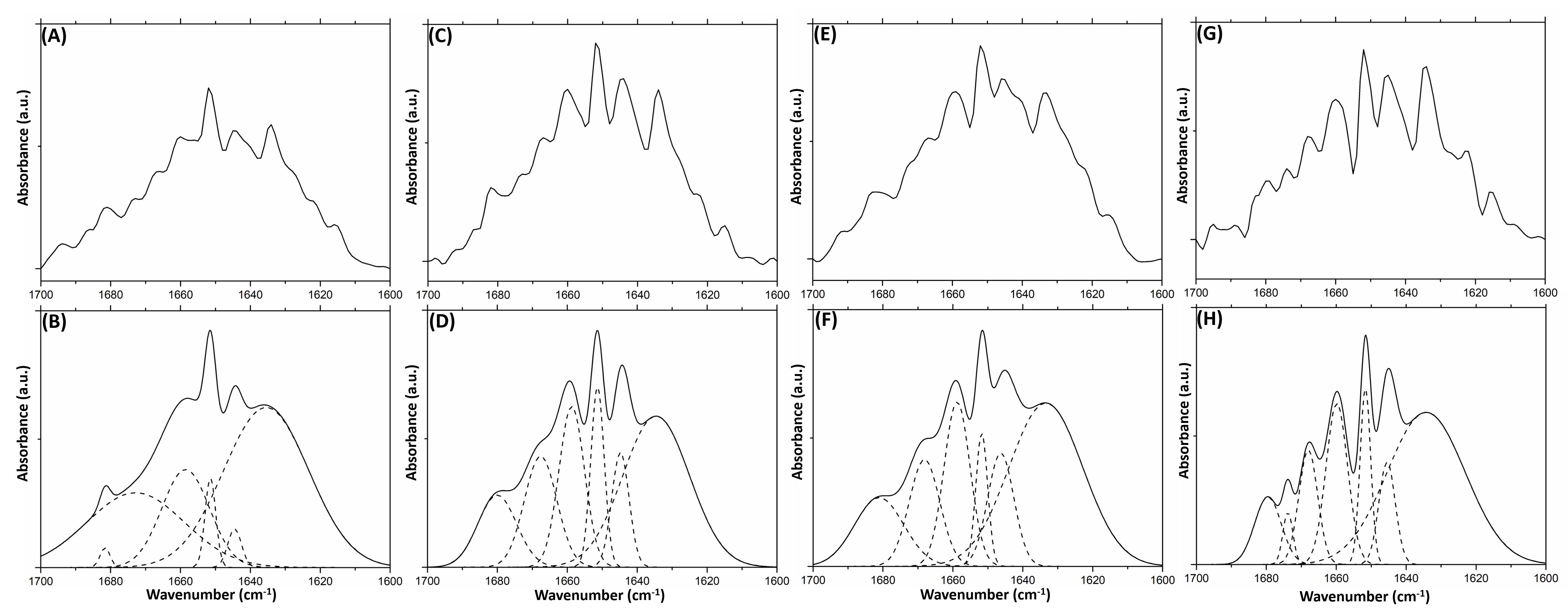

Supplement: Supplementary file 5 — Additional file 5: Figure S4. (A) Representative mean spectra of Tet(X2) protein solution shown in the range 1600–1700 cm-1 after baseline correction and vectorial normalization; (B) Curve-fitting analysis of amide I band of Tet(X2) protein solution; (C) Representative mean spectra of Tet(X4) protein solution shown in the range 1600–1700 cm-1 after baseline correction and vectorial normalization; (D) Curve-fitting analysis of amide I band of Tet(X4) ; (E) Representative mean spectra of the L282S mutant protein solution shown in the range 1600–1700 cm-1 after baseline correction and vectorial normalization; (F) Curve-fitting analysis of amide I band of the L282S mutant protein; (G) Representative mean spectra of the V329M mutant protein solution shown in the range 1600–1700 cm-1 after baseline correction and vectorial normalization; (H) Curve-fitting analysis of amide I band of the V329M mutant protein. [file 12915_2021_1199_MOESM5_ESM.tif]

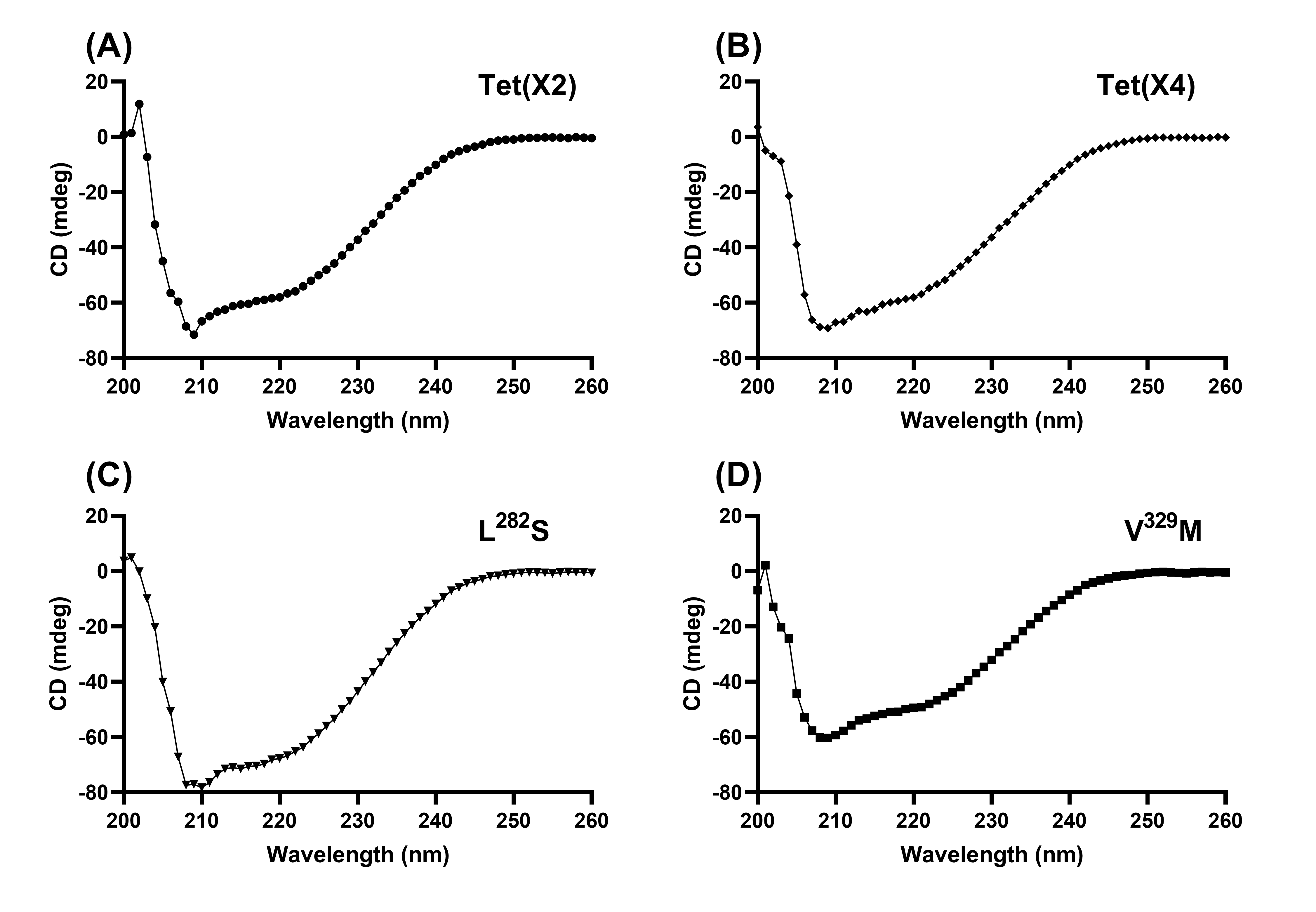

Supplement: Supplementary file 6 — Additional file 6: Figure S5. (A) Circular dichroic spectral profiles of Tet(X2) protein solution measured at wavelength between 200 nm and 260 nm; the raw CD spectrum is shown; (B) Circular dichroic spectral profiles of Tet(X4) protein solution measured at wavelength between 200 nm and 260 nm; (C) Circular dichroic spectral profiles of the L282S mutant protein solution measured at wavelength between 200 nm and 260 nm; (D) Circular dichroic spectral profiles of the V329M mutant protein solution measured at wavelength between 200 nm and 260 nm. [file 12915_2021_1199_MOESM6_ESM.tif]
